# Associations between diet and mental health using the 12-item General Health Questionnaire: cross-sectional and prospective analyses from the Japan Multi-Institutional Collaborative Cohort Study

**DOI:** 10.1186/s12937-019-0515-6

**Published:** 2020-01-09

**Authors:** Naoki Choda, Kenji Wakai, Mariko Naito, Nahomi Imaeda, Chiho Goto, Kenta Maruyama, Yuka Kadomatsu, Mineko Tsukamoto, Tae Sasakabe, Yoko Kubo, Rieko Okada, Sayo Kawai, Takashi Tamura, Asahi Hishida, Kenji Takeuchi, Atsuyoshi Mori, Nobuyuki Hamajima

**Affiliations:** 1 Department of Preventive Medicine, Nagoya University Graduate School of Medicine, Nagoya, Aichi 466-8550 Japan; 2 Department of Oral Epidemiology, Hiroshima University Graduate School of Biomedical and Health Sciences, Hiroshima, 734-8553 Japan; 3 Department of Nutrition, Faculty of Wellness, Shigakkan University, Obu, Aichi 474-8651 Japan; 4 Department of Health and Nutrition, Nagoya Bunri University, Inazawa, Aichi 492-8520 Japan; 5 Department of Public Health, Aichi Medical University School of Medicine, Nagakute, Aichi 480-1195 Japan; 6 Seirei Preventive Health Care Center, Hamamatsu, Shizuoka 433-8105 Japan; 7 Department of Healthcare Administration, Nagoya University Graduate School of Medicine, Nagoya, Aichi 466-8550 Japan

**Keywords:** Mental health, General Health Questionnaire, Nutrition, Food, Calcium, Japanese, Cohort studies

## Abstract

**Background:**

Mental health has become a major public health issue worldwide. Biological and epidemiological studies suggest diet has a role in the prevention or cure of mental disorders. However, further research is required to elucidate the relationship between diet and mental health. This study aimed to investigate associations between dietary intake of nutrients (macronutrients, vitamins, calcium, and fatty acids) and food groups (fish, meat and chicken, dairy products, and vegetables) and mental health among middle-aged Japanese in cross-sectional and prospective studies.

**Methods:**

In total, 9298 men and women that participated in two areas of the Japan Multi-Institutional Collaborative Cohort Study were eligible for analysis at the baseline (cross-sectional) survey. Of these, 4701 participants were followed for about 5 years and included in the follow-up (prospective) analysis. The 12-item General Health Questionnaire (GHQ) was used to assess participants’ general mental health status over the past several weeks. The average intake of 46 foods over the past year was assessed by a validated food frequency questionnaire. We also evaluated lifestyle and medical factors using a self-administered questionnaire. A cross-sectional logistic regression analysis was performed to estimate odds ratios for a GHQ score ≥ 4 (poor mental health) according to dietary intake of foods/nutrients at baseline. The prospective study used baseline dietary and lifestyle factors and GHQ scores at follow-up.

**Results:**

The cross-sectional logistic regression analysis showed vegetables, protein, calcium, vitamin D, carotene and n-3 highly-polyunsaturated fatty acids were inversely associated with a GHQ score ≥ 4. On the other hand, mono-unsaturated fatty acids showed a positive association with higher GHQ score. The prospective logistic regression analysis found dairy products, calcium, vitamin B_2_, and saturated fatty acids were inversely correlated with a GHQ score ≥ 4. Calcium was associated with GHQ scores in both the cross-sectional and follow-up studies. In the follow-up study, the multivariable-adjusted odds ratio for a GHQ score ≥ 4 was 0.71 (95% confidence interval, 0.55–0.92) for the highest versus lowest quartiles of calorie-adjusted dietary calcium intake.

**Conclusion:**

Consuming particular nutrients and foods, especially calcium and dairy products, may lead to better mental health in Japanese adults.

## Background

Mental health has become a major public health issue worldwide. It has been estimated that mental disorders (represented by depressive and anxiety disorders) account for 13% of the global burden of disease in terms of disability-adjusted life-years, which is equivalent to cardiovascular and circulatory diseases [1]. Mental disorders are also common in Japan, with the prevalence of common mental disorders (e.g. major depression, specific phobia, and alcohol abuse/dependence) reported to be 8.8% [2]. Furthermore, a patient survey conducted by the Japan Ministry of Health, Labour and Welfare showed the number of patients with mental disorders in Japan had almost doubled in the last two decades, from 2 million in 2000 to 4 million in 2015.

It is important to address the increasing prevalence of mental disorders because these disorders reduce people’s quality of life, worsen many medical illnesses, promote disability, and increase mortality [3]. Furthermore, mental disorders can lead to other social problems, such as loss of productivity at work, suicide, or criminal behavior [4–6]. Therefore, mental disorders impose a major burden on individuals and society.

There has been a steady increase in biological and epidemiological studies investigating the relationships between diet and mental health [7, 8]. It has been suggested that various dietary patterns, foods, and nutrients may affect the onset, prolongation, and severity of mental disorders. For example, a positive association was found between Western dietary patterns and mental disorders such as depression and anxiety, whereas Mediterranean dietary patterns were reported to have protective effects [7, 9, 10]. High intakes of fruit, vegetables, fish, poultry, dairy products, unprocessed meat, and whole grains have been associated with a reduced depression risk [8, 11, 12]. Many nutrients are suggested to be effective against depression, including calcium, magnesium, zinc, folate, vitamins D and B_12_, and n-3 polyunsaturated fatty acids (PUFA) [8, 13].

Although diet may have a role in the prevention or cure of mental disorders, especially depression, further research is needed to elucidate the diet-mental health relationship. Some challenges and unclear points have been highlighted. First, although prospective (longitudinal) studies can reflect causal relationships, there are fewer prospective reports available than cross-sectional studies. Second, from the perspective of explanatory variables, analyzing dietary patterns or foods tells us how/what to eat; however, key components remain unclear, which may be detected by analysis of nutrients. Finally, even though the 12-item General Health Questionnaire (GHQ-12) is widely used for screening common and general mental disorders not limited to specific diseases [14], few reports have used the GHQ-12 compared with other scales specific to depression, such as the Center for Epidemiological Studies Depression Scale. Therefore, this study aimed to explore the associations between dietary intake of food groups/nutrients and general mental health assessed with the GHQ-12 in a Japanese population, using both cross-sectional and prospective studies. The food groups included fish, meat and chicken, dairy products, and vegetables. We also investigated three macronutrients, vitamins, calcium, and fatty acids.

## Methods

This study used the STROBE-Nut as a reporting guideline (Additional file 1).

### The Japan Multi-Institutional Collaborative Cohort (J-MICC) Study

The J-MICC Study is a cohort study that aimed to elucidate the interactions among genotypes, lifestyles, and lifestyle-related diseases, especially cancer [15]. The J-MICC Study was launched in 2005 and plans to follow participants until 2025. We conducted the present analyses in two areas covered by the J-MICC Study: the Daiko Study and the Shizuoka area, because GHQ-12 data were available in these two areas.

### Participants

In the Daiko Study, Nagoya city residents aged 35–69 years were invited to participate in the baseline survey between 2008 and 2010 through posted leaflets [16]. When the dataset was fixed for analysis in May 2015, 21 of the 5174 participants had withdrawn from the study or were ineligible to participate; therefore, 5153 men and women were available for the baseline analyses. Approximately 5 years later (2013–2016), 3543 participants (68.8%) took part in a second (follow-up) survey.

In the Shizuoka area of the J-MICC Study, participants were residents aged 35–69 years who attended the Seirei Preventive Health Care Center in Hamamatsu for a health check-up [17]. Among 13,740 examinees that met the eligibility criteria, 5040 (36.7%) were enrolled between 2006 and 2007. When the dataset was fixed for analysis in May 2015, 32 participants had withdrawn from the study or were ineligible to participate, leaving 5008 available for baseline analyses. Approximately 6 years later (2012–2013), 3746 participants (74.8%) responded to the second (follow-up) survey.

**Fig. 1:**
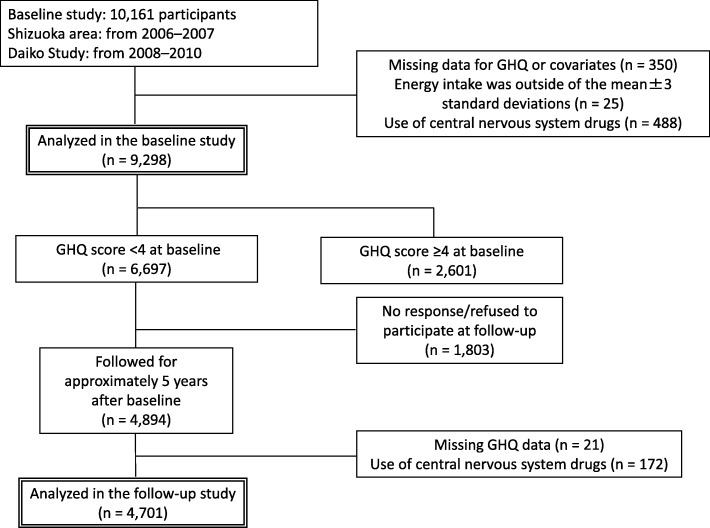
Flowchart of the study protocol. GHQ: General Health Questionnaire

In total, 9298 participants met the eligibility criteria and were included in the baseline analyses. We followed 6697 participants whose GHQ score was < 4 at baseline; 4701 were included in the follow-up study after excluding dropouts and ineligible participants. In the baseline and follow-up studies, we excluded participants who had missing data on GHQ score or covariates, those who reported extreme total energy intake (outside the mean ± 3 standard deviations [SD]) and those who regularly took medication that may affect the central nervous system. The flowchart of the study protocol is presented in Fig. 1.

### Mental health assessment

We used the GHQ-12 to assess participants’ general mental health status over the past several weeks. The GHQ-12 is a widely used scale for measuring general psychological well-being. According to the factor structure analysis in a Japanese adult population, the GHQ-12 is a valid scale that mainly produces psychological distress and social dysfunction factors [18]. Each GHQ-12 item describes a particular behavior or symptom. There are four possible responses to each item: not at all, no more than usual, rather more than usual, or much more than usual. The first and second responses are scored as zero, and the third and fourth responses are scored as 1. The individual item scores are summed, giving a total score from 0 to 12. A GHQ-12 score ≥ 4 is considered to reflect poor mental health [19].

### Dietary assessment

The average intake of 46 foods over the past year was assessed with a validated short food frequency questionnaire (FFQ), as described in previous reports [20–23]. For daily amounts of three staple foods (rice, bread, and noodles), questions were asked about the portion/serving size and frequency of consumption (six categories: almost none, 1–3 times/month, 1–2 times/week, 3–4 times/week, 5–6 times/week, and daily). For the other 43 dietary items, we only investigated intake frequency (eight categories: almost none, 1–3 times/month, 1–2 times/week, 3–4 times/week, 5–6 times/week, 1 time/day, 2 times/day, and ≥ 3 times/day) [22]. The dietary intakes of foods/nutrients were estimated using a program developed and validated at the Department of Public Health, Nagoya City University School of Medicine, based on the standard tables of food composition in Japan (fifth revised edition) [22, 24].

### Other variables

We evaluated lifestyle factors (smoking habits, alcohol consumption, sleeping time, physical activity, and eating breakfast), educational attainment, employment status and medicine intake using a self-administered questionnaire [15]. Employment status was dichotomized based on participants’ status at baseline (working, not working). Smoking habits and alcohol consumption were both divided into five categories (for smoking: never, former, current smoker of < 20 cigarettes/day, 20–39 cigarettes/day, ≥ 40 cigarettes/day; for drinking: never, former, current drinker of < 150 g/week, 150–300 g/week, and ≥ 300 g/week). Participants were defined as current drinkers if they drank once a month or more frequently. We also asked how many hours participants slept per day and how many times they ate breakfast per week. Physical activity was assessed in terms of metabolic equivalents (METs) of leisure-time activity, as reported elsewhere [25]. Participants’ highest educational attainment was also covered in the baseline survey. Educational attainment was classified using three groups: primary/secondary education, tertiary education (undergraduate/graduate school), and others (college of technology, vocational school, community college). For medicine intake, we asked whether participants were regularly taking six types of medicine, including sleeping medication. In addition, they were requested to list any medicines they regularly took that were not classified in these six categories. We checked the intakes of sleeping drugs, anti-anxiety agents, anti-depressant drugs, and anti-psychotic drugs. We did not consider other central nervous system drugs, such as anti-epileptic agents and anti-dementia drugs.

### Statistical analysis

Differences in baseline characteristics between participants with a GHQ score < 4 and those with a GHQ score ≥ 4 were assessed using *t*-tests (for continuous variables) or *χ*^*2*^ tests (for categorical variables). Differences in food or nutrient intakes between the two groups were assessed with the Mann-Whitney test (foods) or *t*-tests after logarithmic conversion (nutrients).

We estimated the odds ratio (OR) for a GHQ score ≥ 4 according to intakes of foods and nutrients using multivariable logistic regression analyses. Consumption of fish, meat, chicken, dairy products, and vegetables was energy-adjusted by dividing total energy intake and was categorized into quartiles. Similarly, we included quartiles of energy-adjusted intakes into the logistic models for: protein; fat; carbohydrate; calcium; vitamins B_1_, B_2_, and D; β-carotene; saturated fatty acids (SFA); monounsaturated fatty acids (MUFA); n-6 PUFA; n-3 PUFA, and n-3 highly-polyunsaturated fatty acids (n-3 HUFA). All values for foods, nutrients, and other covariates were measured at baseline. Confounding factors adjusted for (as categorical variables) were: study area (Daiko or Shizuoka), sex (men or women), current employment status (employed or not employed), current smoking status (five categories), and current drinking status (five categories). Age (years), sleeping time (h/day), leisure time exercise (METs h/day), eating breakfast (times/week), and total energy (kcal/day) were adjusted as continuous variables.

In the prospective study, we followed those whose GHQ score was < 4 at baseline. The OR for a GHQ score ≥ 4 at the follow-up study by baseline intakes of foods and nutrients was estimated by using a logistic model adjusting for the same covariates as in the cross-sectional study. Trends in associations (*P*-trend) across quartiles of food group or nutrient intake were assessed using multivariable logistic regression analysis, with median values assigned to each quartile category.

For sensitivity analyses, we analyzed data including participants taking central nervous system drugs. We also calculated the ORs for calcium and SFA by further adjusting for dairy products in the prospective study, and assessed the influence of further adjustment for educational attainment. In the prospective study, we added analysis without excluding those with GHQ score ≥ 4 at baseline. Furthermore, to consider the changes in calcium intake from baseline to follow-up, we created four groups using the median value of the calorie-adjusted calcium intake at baseline and follow-up as cutoff values: maintained high intake, increased intake, decreased intake, and maintained low intake (reference). The OR for GHQ score ≥ 4 at follow-up was calculated after adjustment for the same covariates as those in the abovementioned model for the cross-sectional study.

In logistic regression analysis, it has been suggested that the number of events per independent variable should be 10 or greater to avoid bias in the estimation of the regression coefficients [26]. The value was 216 at baseline and 65 at follow-up. We could include therefore many more participants in the analyses.

All analyses used SPSS version 24.0 for Windows (IBM, Armonk, NY) and SAS version 9.1 for Windows (SAS Institute, Cary, NC).

## Results

Of the 9298 eligible participants at baseline, 2601 (27.8%) showed poor mental health (GHQ score ≥ 4) (Table 1). The 6697 participants with a GHQ score < 4 at baseline were followed, and 4701 (70.2%) were analyzed in the follow-up study after excluding dropouts (*n* = 1803) and ineligible participants (*n* = 193). In total, 783 of the 4701 participants (16.7%) showed poor mental health at follow-up (Table 2).

**Table 1:** Characteristics of participants at baseline (*n* = 9298)

	Participants with a GHQ score ≥ 4		Participants with a GHQ score < 4		*P* ^a^
Categorical variables	n	%	n	%	
Number of participants	2601		6697		
Area (Shizuoka)	1084	41.7	3578	53.4	< 0.001
Sex (women)	1543	59.3	3227	48.2	< 0.001
Current worker	1996	76.7	4987	74.5	0.023
Smoking					0.002
Never	1623	62.4	3889	58.1	
Former	619	23.8	1812	27.1	
Current < 20 cigarettes/day	178	6.8	496	7.4	
Current 20–39 cigarettes/day	161	6.2	462	6.9	
Current ≥40 cigarettes/day	20	0.8	38	0.6	
Drinking					0.001
Never	1073	41.3	2720	40.6	
Former	49	1.9	82	1.2	
Current < 150 g/week	1026	39.4	2500	37.3	
Current 150–300 g/week	260	10.0	812	12.1	
Current ≥300 g/week	193	7.4	583	8.7	
Continuous variables	Mean	SD	Mean	SD	
Age (years)	50.1	9.1	52.9	9.5	< 0.001
Sleeping time (h/day)	6.4	0.9	6.6	0.9	< 0.001
Leisure-time exercise (METs • h/day)	1.6	2.6	2.0	2.9	< 0.001
Eating breakfast (times/week)	6.3	1.6	6.5	1.4	< 0.001

*GHQ* General Health Questionnaire, *METs* Metabolic equivalents, *SD* Standard deviation

^a^Categorical variables: χ^2^ test; continuous variables: *t*-test

**Table 2:** Participants’ (baseline) characteristics at follow-up (*n* = 4701)

	Participants with a GHQ score ≥4		Participants with a GHQ score <4		*P* ^a^
Categorical variables	n	%	n	%	
Number of participants	783		3918		
Area (Shizuoka)	433	55.3	2173	55.5	0.934
Sex (women)	432	55.2	1805	46.1	< 0.001
Current worker	609	77.8	2918	74.5	0.051
Smoking					0.047
Never	496	63.3	2285	58.3	
Former	186	23.8	1123	28.7	
Current < 20 cigarettes/day	47	6.0	266	6.8	
Current 20–39 cigarettes/day	50	6.4	229	5.8	
Current ≥40 cigarettes/day	4	0.5	15	0.4	
Drinking					0.21
Never	349	44.6	1575	40.2	
Former	9	1.1	42	1.1	
Current < 150 g/week	283	36.1	1498	38.2	
Current 150–300 g/week	87	11.1	475	12.1	
Current ≥300 g/week	55	7.0	328	8.4	
Continuous variables	Mean	SD	Mean	SD	
Age (years)	50.2	9.1	54.0	9.1	< 0.001
Sleeping time (h/day)	6.5	0.9	6.7	0.9	< 0.001
Leisure-time exercise (METs ▪ h/day)	1.8	2.5	2.2	3.0	< 0.001
Eating breakfast (times/week)	6.5	1.3	6.6	1.3	0.168

*GHQ* General Health Questionnaire, *METs* Metabolic equivalents, *SD* Standard deviation

^a^Categorical variables: χ^2^ test; continuous variables: *t*-test

Approximately half of the participants at both baseline and follow-up were women. At both measurements, the proportion of males, proportion of elder people, sleeping time, and leisure-time exercise were significantly lower among participants with a GHQ score ≥ 4 than those with a GHQ score < 4. Tables 3 and 4 show food and nutrient intakes at baseline and follow-up, respectively. Intakes of fish, calcium, vitamins B_2_ and D, and n-3 HUFA were significantly lower in those with a GHQ score ≥ 4 than those with a GHQ score < 4 at both baseline and follow-up. In contrast, those with a GHQ score ≥ 4 consumed significantly more fat, MUFA, and n-6 PUFA than those with a GHQ-12 score < 4.

**Table 3:** Daily intake of food groups and nutrients among participants at baseline (*n* = 9298)

Continuous variables	Participants with a GHQ score ≥ 4		Participants with a GHQ score < 4		
	Mean	SD	Mean	SD	*P*^a^
Food groups (g)					
Fish	46.0	24.8	50.2	26.9	< 0.001
Meat and chicken	38.3	21.7	37.2	21.6	0.006
Dairy products	123.2	104.5	126.4	105.3	0.147
Vegetables	132.9	77.9	136.9	78.7	0.012
Nutrients					
Energy (kcal)	1667	330	1718	338	< 0.001
Protein (g)	51.7	10.2	53.2	10.7	< 0.001
Fat (g)	44.5	10.6	43.7	10.8	0.001
Carbohydrate (g)	232	60	242	62	< 0.001
Calcium (mg)	510	141	523	143	< 0.001
Vitamin B_1_ (mg)	0.654	0.081	0.652	0.086	0.192
Vitamin B_2_ (mg)	1.08	0.25	1.10	0.25	< 0.001
Vitamin D (μg)	6.69	2.84	7.30	3.20	< 0.001
Carotene (μg)	3113	1381	3170	1334	0.009
Saturated fatty acids (g)	11.41	2.67	11.36	2.70	0.409
Monounsaturated fatty acids (g)	16.40	3.84	16.09	3.81	< 0.001
n-6 polyunsaturated fatty acids (g)	11.07	2.90	10.91	2.87	0.012
n-3 polyunsaturated fatty acids (g)	2.22	0.53	2.23	0.55	0.250
n-3 highly-polyunsaturated fatty acids (g)	0.664	0.282	0.720	0.318	< 0.001

*GHQ* General Health Questionnaire, *SD* Standard deviation. ^a^Food groups: Mann-Whitney test; nutrients: *t*-test after logarithmic transformation

**Table 4:** Daily intake of food groups and nutrients at baseline among participants at follow-up (*n* = 4701)

Continuous variables	Participants with a GHQ score ≥ 4		Participants with a GHQ score < 4		
	Mean	SD	Mean	SD	*P*^a^
Food groups (g)					
Fish	49.9	31.0	50.9	25.8	0.009
Meat and chicken	38.9	25.3	36.9	21.3	0.080
Dairy products	125.3	109.1	132.2	107.2	0.023
Vegetables	141.9	78.2	138.5	79.4	0.136
Nutrients					
Energy (kcal)	1715	350	1727	327	0.193
Protein (g)	53.5	12.0	53.5	10.3	0.546
Fat (g)	45.0	11.8	43.8	10.6	0.013
Carbohydrate (g)	241	64	244	61	0.131
Calcium (mg)	521	148	532	145	0.038
Vitamin B_1_ (mg)	0.656	0.098	0.651	0.083	0.232
Vitamin B_2_ (mg)	1.10	0.25	1.12	0.25	0.006
Vitamin D (μg)	7.22	3.60	7.41	3.15	0.019
Carotene (μg)	3257	1394	3197	1351	0.244
Saturated fatty acids (g)	11.48	3.01	11.43	2.69	0.997
Monounsaturated fatty acids (g)	16.58	4.16	16.05	3.76	0.001
n-6 polyunsaturated fatty acids (g)	11.32	3.03	10.90	2.83	< 0.001
n-3 polyunsaturated fatty acids (g)	2.29	0.61	2.24	0.54	0.060
n-3 highly-polyunsaturated fatty acids (g)	0.714	0.364	0.730	0.314	0.032

*GHQ* General Health Questionnaire, *SD* Standard deviation. ^a^Food groups: Mann-Whitney test; nutrients: *t*-test after logarithmic transformation

Table 5 shows the results of the cross-sectional multiple logistic regression analyses using baseline data. We observed inverse associations between a GHQ score ≥ 4 and protein, calcium, vitamin D, carotene, and n-3 HUFA (*P*-trend < 0.05), whereas MUFA showed a positive correlation (Model 2). Vegetables demonstrated a significant inverse association and dairy products showed a marginally insignificant negative correlation with a GHQ score ≥ 4 (*P*-trend = 0.059).

**Table 5:** Cross-sectional logistic regression analyses for the association between food/nutrient intake and GHQ-12 score

		Participants with GHQ score ≥ 4 (n)	Model 1 (OR, 95% CI)^a^		*P*-trend	Model 2 (OR, 95% CI)^b^		*P*-trend
Food groups								
Fish	Q1 (low)	707	1	(Ref.)	0.064	1	(Ref.)	0.070
	Q2	701	0.98	(0.86–1.11)		0.97	(0.85–1.10)	
	Q3	598	0.84	(0.74–0.96)		0.84	(0.73–0.96)	
	Q4 (high)	595	0.91	(0.79–1.04)		0.90	(0.79–1.04)	
Meat and chicken	Q1 (low)	564	1	(Ref.)	0.876	1	(Ref.)	0.977
	Q2	633	0.99	(0.86–1.13)		0.99	(0.87–1.14)	
	Q3	683	1.01	(0.88–1.16)		1.01	(0.88–1.16)	
	Q4 (high)	721	1.00	(0.87–1.15)		1.00	(0.87–1.15)	
Dairy products	Q1 (low)	652	1	(Ref.)	0.011	1	(Ref.)	0.059
	Q2	647	0.97	(0.85–1.11)		0.99	(0.87–1.14)	
	Q3	661	0.97	(0.85–1.11)		1.00	(0.87–1.14)	
	Q4 (high)	641	0.84	(0.74–0.96)		0.88	(0.77–1.01)	
Vegetables	Q1 (low)	660	1	(Ref.)	0.026	1	(Ref.)	0.045
	Q2	635	0.84	(0.74–0.96)		0.86	(0.75–0.98)	
	Q3	662	0.85	(0.74–0.98)		0.87	(0.75–1.00)	
	Q4 (high)	644	0.82	(0.71–0.95)		0.83	(0.72–0.96)	
Nutrients								
Protein	Q1 (low)	628	1	(Ref.)	0.002	1	(Ref.)	0.003
	Q2	662	0.95	(0.83–1.09)		0.94	(0.82–1.07)	
	Q3	656	0.85	(0.74–0.97)		0.84	(0.73–0.97)	
	Q4 (high)	655	0.82	(0.71–0.94)		0.82	(0.71–0.95)	
Carbohydrate	Q1 (low)	542	1	(Ref.)	0.571	1	(Ref.)	0.996
	Q2	621	0.88	(0.78–1.00)		0.92	(0.80–1.05)	
	Q3	688	0.85	(0.75–0.97)		0.88	(0.76–1.01)	
	Q4 (high)	750	1.00	(0.87–1.14)		1.04	(0.88–1.22)	
Fat	Q1 (low)	746	1	(Ref.)	0.641	1	(Ref.)	0.980
	Q2	663	1.02	(0.88–1.17)		0.99	(0.86–1.15)	
	Q3	590	1.01	(0.88–1.17)		1.00	(0.86–1.16)	
	Q4 (high)	602	1.04	(0.89–1.21)		1.00	(0.85–1.17)	
Calcium	Q1 (low)	630	1	(Ref.)	0.005	1	(Ref.)	0.006
	Q2	668	0.98	(0.86–1.12)		0.94	(0.82–1.08)	
	Q3	648	0.87	(0.76–1.00)		0.85	(0.74–0.99)	
	Q4 (high)	655	0.83	(0.72–0.96)		0.82	(0.70–0.95)	
Vitamin B_1_	Q1 (low)	525	1	(Ref.)	0.430	1	(Ref.)	0.785
	Q2	642	1.09	(0.95–1.25)		1.07	(0.92–1.24)	
	Q3	706	1.10	(0.94–1.27)		1.05	(0.88–1.24)	
	Q4 (high)	728	1.08	(0.93–1.26)		1.00	(0.82–1.22)	
Vitamin B_2_	Q1 (low)	633	1	(Ref.)	0.138	1	(Ref.)	0.098
	Q2	625	0.91	(0.80–1.04)		0.90	(0.79–1.04)	
	Q3	693	0.99	(0.86–1.13)		0.98	(0.84–1.13)	
	Q4 (high)	650	0.87	(0.76–1.01)		0.85	(0.73–1.00)	
Vitamin D	Q1 (low)	694	1	(Ref.)	0.008	1	(Ref.)	0.009
	Q2	719	0.98	(0.86–1.12)		0.95	(0.84–1.09)	
	Q3	606	0.84	(0.73–0.96)		0.83	(0.72–0.95)	
	Q4 (high)	582	0.86	(0.75–0.99)		0.85	(0.74–0.98)	
Carotene	Q1 (low)	651	1	(Ref.)	0.010	1	(Ref.)	0.015
	Q2	654	0.87	(0.76–0.99)		0.86	(0.75–0.99)	
	Q3	640	0.78	(0.68–0.90)		0.79	(0.68–0.91)	
	Q4 (high)	656	0.81	(0.70–0.93)		0.80	(0.69–0.94)	
SFA	Q1 (low)	563	1	(Ref.)	0.180	1	(Ref.)	0.097
	Q2	643	1.00	(0.87–1.15)		0.97	(0.84–1.12)	
	Q3	672	0.92	(0.79–1.07)		0.88	(0.76–1.03)	
	Q4 (high)	722	0.92	(0.78–1.07)		0.88	(0.75–1.04)	
MUFA	Q1 (low)	518	1	(Ref.)	0.001	1	(Ref.)	0.019
	Q2	635	1.12	(0.97–1.29)		1.11	(0.96–1.28)	
	Q3	678	1.11	(0.96–1.28)		1.10	(0.95–1.28)	
	Q4 (high)	770	1.28	(1.10–1.48)		1.22	(1.04–1.42)	
n-6 PUFA	Q1 (low)	546	1	(Ref.)	0.040	1	(Ref.)	0.130
	Q2	644	1.07	(0.93–1.22)		1.04	(0.91–1.20)	
	Q3	660	1.02	(0.88–1.17)		1.00	(0.87–1.16)	
	Q4 (high)	751	1.17	(1.02–1.35)		1.13	(0.97–1.31)	
n-3 PUFA	Q1 (low)	614	1	(Ref.)	0.119	1	(Ref.)	0.434
	Q2	600	0.85	(0.74–0.97)		0.84	(0.73–0.96)	
	Q3	668	0.95	(0.83–1.09)		0.91	(0.79–1.05)	
	Q4 (high)	719	1.05	(0.92–1.21)		1.00	(0.86–1.16)	
n-3 HUFA	Q1 (low)	687	1	(Ref.)	0.020	1	(Ref.)	0.018
	Q2	713	0.96	(0.85–1.10)		0.93	(0.81–1.06)	
	Q3	616	0.88	(0.77–1.01)		0.87	(0.76–1.00)	
	Q4 (high)	585	0.86	(0.75–0.99)		0.85	(0.73–0.98)	

*GHQ* General Health Questionnaire, *CI* Confidence interval, *OR* Odds ratio, *Q1–Q4* Quartiles 1–4, *SFA* Saturated fatty acids, *MUFA* Monounsaturated fatty acids, *PUFA* Polyunsaturated fatty acids, *HUFA* Highly-polyunsaturated fatty acids

Odds ratios shown by quartile of intake (*n* = 9298)

^a^Model 1: adjusted for sex, age, and area

^b^Model 2: adjusted for sex, age, area, employment, smoking, drinking, sleeping time, leisure time exercise, eating breakfast, and total energy

Table 6 summarizes the result of the prospective multiple logistic regression analyses using follow-up data. Dairy products, calcium, vitamin B_2_, and SFA showed inverse associations (*P*-trend < 0.05) with a GHQ score ≥ 4, even after adjustment for covariates (Model 2). Calcium showed an association in both the cross-sectional and prospective studies. The multivariable-adjusted OR for a GHQ score ≥ 4 was 0.71 (95% confidence interval [CI], 0.55–0.92) in the highest versus lowest quartiles of calorie-adjusted dietary calcium intake at follow-up (Table 6).

**Table 6:** Prospective logistic regression analyses for association between food/nutrient intake and GHQ-12 score*

		Participants with GHQ score ≥ 4 (n)	Model 1 (OR, 95% CI)^a^		*P*-trend	Model 2 (OR, 95% CI)^b^		*P*-trend
Food groups								
Fish	Q1 (low)	211	1	(Ref.)	0.757	1	(Ref.)	0.886
	Q2	203	0.94	(0.75–1.16)		0.94	(0.75–1.16)	
	Q3	194	0.97	(0.78–1.21)		0.98	(0.79–1.22)	
	Q4 (high)	175	0.95	(0.75–1.19)		0.97	(0.77–1.22)	
Meat and chicken	Q1 (low)	186	1	(Ref.)	0.345	1	(Ref.)	0.332
	Q2	191	0.84	(0.67–1.06)		0.87	(0.69–1.10)	
	Q3	183	0.76	(0.60–0.96)		0.78	(0.61–0.98)	
	Q4 (high)	223	0.86	(0.68–1.08)		0.87	(0.69–1.09)	
Dairy products	Q1 (low)	208	1	(Ref.)	0.002	1	(Ref.)	0.002
	Q2	216	1.04	(0.84–1.29)		1.04	(0.83–1.29)	
	Q3	176	0.75	(0.60–0.94)		0.74	(0.59–0.93)	
	Q4 (high)	183	0.75	(0.59–0.94)		0.75	(0.59–0.95)	
Vegetables	Q1 (low)	173	1	(Ref.)	0.362	1	(Ref.)	0.291
	Q2	212	1.17	(0.93–1.47)		1.20	(0.95–1.50)	
	Q3	183	0.96	(0.76–1.22)		0.98	(0.77–1.25)	
	Q4 (high)	215	1.18	(0.92–1.51)		1.21	(0.95–1.55)	
Nutrients								
Protein	Q1 (low)	210	1	(Ref.)	0.082	1	(Ref.)	0.128
	Q2	184	0.77	(0.62–0.96)		0.78	(0.62–0.98)	
	Q3	181	0.69	(0.55–0.87)		0.69	(0.55–0.88)	
	Q4 (high)	208	0.81	(0.64–1.02)		0.83	(0.65–1.05)	
Carbohydrate	Q1 (low)	203	1	(Ref.)	0.307	1	(Ref.)	0.499
	Q2	205	1.07	(0.86–1.33)		1.07	(0.85–1.34)	
	Q3	194	1.11	(0.89–1.39)		1.10	(0.86–1.40)	
	Q4 (high)	181	1.12	(0.88–1.42)		1.09	(0.83–1.43)	
Fat	Q1 (low)	175	1	(Ref.)	0.993	1	(Ref.)	0.601
	Q2	197	0.98	(0.78–1.24)		1.01	(0.80–1.28)	
	Q3	185	0.79	(0.62–1.01)		0.82	(0.64–1.06)	
	Q4 (high)	226	0.92	(0.72–1.19)		0.96	(0.74–1.25)	
Calcium	Q1 (low)	216	1	(Ref.)	0.005	1	(Ref.)	0.019
	Q2	188	0.77	(0.62–0.97)		0.79	(0.63–0.99)	
	Q3	191	0.73	(0.58–0.93)		0.75	(0.59–0.96)	
	Q4 (high)	188	0.68	(0.54–0.88)		0.71	(0.55–0.92)	
Vitamin B_1_	Q1 (low)	177	1	(Ref.)	0.395	1	(Ref.)	0.878
	Q2	192	0.92	(0.73–1.17)		0.99	(0.77–1.28)	
	Q3	199	0.83	(0.65–1.07)		0.91	(0.68–1.22)	
	Q4 (high)	215	0.89	(0.68–1.16)		1.02	(0.74–1.42)	
Vitamin B_2_	Q1 (low)	213	1	(Ref.)	0.001	1	(Ref.)	0.007
	Q2	198	0.83	(0.66–1.03)		0.84	(0.67–1.05)	
	Q3	188	0.72	(0.57–0.91)		0.73	(0.58–0.94)	
	Q4 (high)	184	0.68	(0.53–0.87)		0.70	(0.54–0.91)	
Vitamin D	Q1 (low)	206	1	(Ref.)	0.607	1	(Ref.)	0.832
	Q2	220	1.00	(0.81–1.25)		1.03	(0.83–1.28)	
	Q3	178	0.87	(0.69–1.09)		0.88	(0.70–1.11)	
	Q4 (high)	179	0.97	(0.77–1.23)		1.01	(0.80–1.28)	
Carotene	Q1 (low)	181	1	(Ref.)	0.863	1	(Ref.)	0.607
	Q2	210	1.06	(0.85–1.33)		1.12	(0.89–1.41)	
	Q3	178	0.84	(0.66–1.07)		0.88	(0.69–1.13)	
	Q4 (high)	214	1.06	(0.82–1.36)		1.12	(0.86–1.44)	
SFA	Q1 (low)	189	1	(Ref.)	0.001	1	(Ref.)	0.003
	Q2	202	0.89	(0.71–1.12)		0.90	(0.71–1.14)	
	Q3	195	0.73	(0.57–0.94)		0.76	(0.59–0.98)	
	Q4 (high)	197	0.65	(0.50–0.85)		0.68	(0.51–0.89)	
MUFA	Q1 (low)	171	1	(Ref.)	0.910	1	(Ref.)	0.688
	Q2	185	0.94	(0.74–1.19)		0.98	(0.77–1.25)	
	Q3	209	0.98	(0.77–1.25)		1.05	(0.82–1.35)	
	Q4 (high)	218	0.96	(0.76–1.23)		1.04	(0.80–1.34)	
n-6 PUFA	Q1 (low)	168	1	(Ref.)	0.624	1	(Ref.)	0.361
	Q2	187	0.98	(0.77–1.23)		1.01	(0.80–1.29)	
	Q3	204	0.99	(0.78–1.25)		1.03	(0.81–1.32)	
	Q4 (high)	224	1.05	(0.82–1.33)		1.11	(0.87–1.43)	
n-3 PUFA	Q1 (low)	174	1	(Ref.)	0.268	1	(Ref.)	0.093
	Q2	198	1.08	(0.86–1.36)		1.14	(0.90–1.44)	
	Q3	192	0.99	(0.79–1.26)		1.06	(0.83–1.35)	
	Q4 (high)	219	1.16	(0.92–1.47)		1.26	(0.99–1.62)	
n-3 HUFA	Q1 (low)	212	1	(Ref.)	0.879	1	(Ref.)	0.853
	Q2	209	0.92	(0.74–1.15)		0.95	(0.76–1.19)	
	Q3	175	0.83	(0.66–1.04)		0.85	(0.67–1.06)	
	Q4 (high)	187	0.99	(0.78–1.24)		1.04	(0.82–1.31)	

All variables (food and nutrient intakes and covariates) were measured at baseline

*GHQ* General Health Questionnaire, *CI* Confidence interval, *OR* Odds ratio, *Q1–Q4* Quartiles 1–4, *SFA* Saturated fatty acids, *MUFA* Monounsaturated fatty acids, *PUFA* Polyunsaturated fatty acids, *HUFA* Highly-polyunsaturated fatty acids

Odds ratios shown by quartile of intake (*n* = 4701)

*Excludes those with a GHQ-12 score ≥ 4 at baseline

^a^Model 1: adjusted for sex, age, and area

^b^Model 2: adjusted for sex, age, area, employment, smoking, drinking, sleeping time, leisure time exercise, eating breakfast, and total energy

To check the role of selective drop-out of participants during follow-up, we examined baseline characteristics of participants with GHQ scores < 4 at baseline (Additional file 2: Table S1). Compared with participants with GHQ scores < 3 at baseline, individuals whose GHQ score was 3 at baseline were more likely to drop out.

Participants might have changed their eating habits during the follow-up period; however, the mean, SD, and quartiles at baseline (Additional file 3: Table S2) did not notably differ from those at follow-up (Additional file 4: Table S3). In addition, Pearson’s correlation coefficients between baseline and follow-up were 0.50 or above for most nutrients (Additional file 4: Table S3).

As a sensitivity analysis, we calculated the OR of calcium and SFA by adjusting for dairy products in the prospective study. The *P* values for trends for calcium and SFA became insignificant after this further adjustment (Additional file 5: Table S4).

In the prospective study, we also analyzed the data without excluding those with GHQ score ≥ 4 at baseline (Additional file 6: Table S5). The overall findings were essentially unchanged from those in Table 6. Dairy products, calcium, vitamin B_2_, and SFA showed negative associations, and *Ps* for trends became smaller. The main difference was that n-3 PUFA showed a significant positive association with GHQ score.

We also conducted an analysis to consider the changes in calcium intake between baseline and follow-up. The multivariable-adjusted OR for a GHQ score ≥ 4 for maintained high calcium intake (number of subjects with GHQ ≥4/total number; 274/1757) versus maintained low calcium intake (308/1770) was 0.78 (95% CI, 0.63–0.97). The OR for increased calcium intake (96/580) or decreased calcium intake (105/594) versus maintained low calcium intake was not statistically significant. The overall findings were not substantially altered when we included participants who were taking central nervous system drugs at baseline or follow-up (data not shown). In addition, further adjustment for educational attainment did not essentially change the findings.

## Discussion

In the present cross-sectional and prospective studies, intakes of several foods and nutrients were inversely associated with high GHQ scores in Japanese adults. Protein showed a significant inverse correlation in the cross-sectional study and a negative association, though with an insignificant linear trend, in the follow-up study. To our knowledge, no previous clinical study has reported an association between dietary intakes of protein and mood in detail. However, protein may be beneficial for mood because tryptophan, an amino acid, is converted to serotonin that plays an important role in mood alleviation, satiety, and sleep regulation [27].

Calcium showed an inverse association with poor mental health both in the cross-sectional and prospective studies. Several investigations have reported similar associations between calcium and depression. A previous meta-analysis reported a pooled relative risk for depression of 0.66 (95% CI, 0.42–1.02) when comparing the highest and lowest intakes [28]. Because that meta-analysis was based on cross-sectional studies, our prospective findings may be meaningful for further understanding the effect of calcium on mood. The biological mechanism underlying the influence of calcium may be activation of tryptophan hydroxylase in serotonin synthesis [29]. However, care is needed with calcium supplementation, as it may be a risk factor for vascular disease [30]. Consistent adverse effects on cardiovascular health have not demonstrated for dietary calcium [31]; therefore, obtaining calcium from the diet rather than supplements should be encouraged.

In our study, the *P*-trend for the inverse correlation with vitamin B_2_ was significant at follow-up and marginally significant at baseline. Some previous reports have also suggested an association between vitamin B_2_ and mental health. Vitamins B_12_, B_6_, and B_2_ may slow the progression of cognitive decline and possibly reduce the risk of depression in ageing [32]. In addition, low intakes of B-vitamins have been related to poor adolescent mental health and behavior [33].

Vitamin D intake has been associated with a lower risk for depression [8]. However, the association in our cross-sectional study might have been due to a reverse causal relationship, as no significant association was observed in the prospective study. Supplementation of β-carotene has been reported to reduce patients’ anxiety and depression scores [34]. However, in our study, carotene showed an inverse association in the cross-sectional study but not in the prospective study.

Increasing evidence from observational studies and randomized placebo-controlled trials regarding fatty acids suggests that n-3 PUFA have ameliorating effects on mood disorders [35]. In laboratory experiments, n-3 PUFA exerted beneficial effects on moods through neuroendocrine modulation or anti-inflammatory effects [36]. However, in our cross-sectional study, n-3 PUFA showed significantly lower OR in the second quartile, and n-3 HUFA in the fourth quartile, and the *P*-trend was only significant for n-3 HUFA. In addition, these associations were not found in the prospective study, meaning the association in the cross-sectional study might have resulted from reverse causality. These unclear associations may be explained by high intakes of n-3 PUFA in Japanese people [37]. A dose-response meta-analysis revealed a J-shaped association with the lowest risk for depression at 1.8 g/d intake of n-3 PUFA [38]. The average daily intake of n-3 PUFA in Japanese men and women aged ≥20 years was reported as 2.47 g and 2.08 g, respectively [39]. In addition, a prospective cohort study in Japanese older adults showed the association with depression was significant; the hazard ratio was lowest in the second quartile for eicosapentaenoic acid and in the third quartile for docosapentaenoic acid, but not significant for total n-3 PUFA [40]. Each component of n-3 PUFA may have maximum effects at different levels and the total effects may be unclear. We could not estimate intakes of individual fatty acids among n-3 PUFA with the FFQ used in this study.

A previous cohort study indicated weak inverse associations between MUFA and depression [41]. MUFA showed a positive association with poor mental health in the present cross-sectional study, but this relationship was not observed in the prospective study. The *P*-trend for SFA was also significant in the prospective study and marginally significant in the cross-sectional study. To our knowledge, there is little evidence to indicate beneficial effects of SFA on mood. The association with SFA might be explained by the intake of dairy products, as no significant association was observed after adjustment for dairy products (Additional file 5: Table S4).

Consumption of dairy products was inversely associated with a GHQ score ≥ 4 in the prospective study and marginally significant in the cross-sectional study. Dairy products contain minerals (e.g., calcium) as well as many vitamins (e.g., vitamins A, D, B_2_, and B_12_), and protein. The association for calcium in the prospective study was attenuated by adjustment for intake of dairy products (Additional file 5: Table S4). This suggests that the effect of dairy products may be partly related to calcium intake or vice versa. Yogurt also contains probiotics, some of which have been reported to be beneficial to depression or anxiety disorders [8]. Some studies have related dairy products to depressive symptoms, but the results were not consistent [42]. This discrepancy may be attributable to different types of dairy products. Interestingly, a previous cross-sectional study involving Japanese adults showed consumption of low-fat dairy (but not whole-fat dairy) was inversely associated with depressive symptoms [42]. Those authors hypothesized that trans-fatty acids in whole-fat milk may be detrimental to depression. However, high consumption of whole-fat yogurt was related to a lower risk for depression in women in a cohort study; the study group speculated that linoleic acid in whole-fat yogurt may have beneficial effects [43]. We did not differentiate low-fat from whole-fat dairy in the FFQ, and therefore could not address this issue.

In our cross-sectional study, vegetable intake showed an inverse association with a GHQ score ≥ 4. This finding may be related to the inverse association between carotene and GHQ score ≥ 4 in the cross-sectional study. Fish, which is rich in n-3 PUFA and protein, showed a somewhat inverse association in the cross-sectional study. A systematic review revealed consumption of fish and vegetables was associated with a somewhat lower incidence of depressive symptoms [9]. However, the reason why the association was not observed in the present prospective study is not clear.

The strength of this study was that it comprehensively examined associations of nutrients and foods with general psychological wellbeing in both cross-sectional and prospective studies and adjusted for several confounders. By analyzing both foods and nutrients, we may have successfully identified the types of foods that are beneficial, as well as key components for better mental health. A cross-sectional study can be conducted in a larger population with greater statistical power than a prospective study. A prospective study can consider incidence and temporality of health problems, but there always is an issue of potential change in participants’ lifestyles during follow-up. Therefore, the significant associations between nutrients (e.g., calcium, protein) and GHQ-12 score in both the cross-sectional and prospective studies suggest more likely relationships compared with findings from either study alone.

About half of the participants in our study were women, and both workers and non-workers were included in the sample. Among those aged 35–69 years at baseline, 23.4% of men (*n* = 4528) and 32.3% of women (*n* = 4770) had a GHQ score ≥ 4. Our study was conducted in only two geographical areas in Japan. However, a previous cross-sectional community-based study in Kanazawa, Japan, reported a GHQ score ≥ 4 in 24.2% of men (*n* = 4693; mean age 51.6 ± 19.5 years) and 32.0% of women (*n* = 5678; mean age 52.4 ± 19.4 years) [44]. These percentages were close to ours, which suggests that our findings may be generalizable to the Japanese adult populations.

Several limitations in this study should be discussed. First, there is potential bias by selective dropout in the prospective study. Those who were more prone to mental health issues or who were actually experiencing mental health problems might have chosen not to participate in the follow-up study. Compared with participants whose GHQ score was < 3 at baseline, participants whose GHQ score was 3 at baseline were more likely to drop out (Additional file 2: Table S1). Participants might have changed their eating habits during the 5 years of follow-up. However, any changes were not drastic, because all Pearson’s correlation coefficients for nutrient intakes between the baseline and follow-up surveys were sufficiently high (Additional file 4: Table S3). Second, although we considered several covariates, unmeasured confounders might still exist. For example, genetic factors may play a role in the pathogenesis of depression [45] or anxiety disorders [46]. Among socioeconomic factors, we only adjusted for employment. However, further adjustment for educational attainment did not alter the findings. In addition, medication or supplementation may be other residual confounders. At baseline, 1599 (17.2%) participants reported regular use of any kind of vitamins or minerals. Furthermore, although we excluded participants who were taking medicine such as anti-depressant drugs, other medicines might have influenced mood [8]. Third, we could not assess the effects of possibly beneficial nutrients on mood (e.g., magnesium and vitamin B_12_), because the FFQ had not been validated for those nutrients. The issue of multiple tests cannot be ruled out, because there were a lot of food groups and nutrients in both cross-sectional and prospective analyses. Our findings need to be confirmed by future studies. Finally, even though the GHQ-12 is widely used for screening common mental disorders [47], it is not a scale for specific mental disorders such as depression. There may be large heterogeneity among individuals with a GHQ score ≥ 4. From another perspective, the use of GHQ score may have an advantage in assessing total impact of dietary factors on several common mental disorders, including depression [47].

Although the effects of diet on individual disorders may not be large, dietary intake may have considerable effects at a population level because of the high prevalence of mental distress as a whole. Diet is modifiable without severe adverse effects and changing dietary habits may be beneficial for prevention and treatment of mental disorders, although further studies are required to confirm our findings.

## Conclusion

The cross-sectional analysis showed intake of vegetables, protein, calcium, vitamin D, carotene, and n-3 HUFA were inversely associated with poor mental health, and intake of MUFA was positively associated. The prospective logistic regression analysis found dairy products, calcium, vitamin B_2_, and SFA were inversely correlated with poor mental health. Consuming adequate amounts of particular nutrients and foods, especially calcium and dairy products, may lead to better mental health in Japanese adults.

## Supplementary information

**Additional file 1:** STROBE Statement—checklist of items that should be included in reports of observational studies; items are boxed if the recommendations are satisfied.

**Additional file 2: Table S1.** Baseline characteristics of participants and those dropping out of the prospective study (*n* = 6697).

**Additional file 3: Table S2.** Cutoff values for quartiles of daily food and nutrient intakes at baseline.

**Additional file 4: Table S3.** Daily intakes of nutrients measured at follow-up (*n* = 4701).

**Additional file 5: Table S4.** Prospective logistic regression analyses of association between calcium/SFAs and GHQ-12 score with adjustment for dairy products.

**Additional file 6: Table S5.** Prospective logistic regression analyses for association between food/nutrient intakes and GHQ-12 score including those with GHQ-12 score ≥ 4 at baseline.

## Data Availability

The datasets generated and analyzed during the present study are not publicly available because we did not obtain informed consent from participants for open use of individual data.

## Abbreviations

CI: Confidence interval
FFQ: Food frequency questionnaire
GHQ: General Health Questionnaire
HUFA: Highly-polyunsaturated fatty acids
J-MICC: Japan Multi-Institutional Collaborative Cohort Study
METs: Metabolic equivalents
MUFA: Monounsaturated fatty acids
OR: Odds ratio
PUFA: Polyunsaturated fatty acids
SD: Standard deviation
SFA: Saturated fatty acids
