# Supplementary material for: Associations between diet and mental health using the 12-item General Health Questionnaire: cross-sectional and prospective analyses from the Japan Multi-Institutional Collaborative Cohort Study
Source: Nutr J. 2020 Jan 9;19:2. doi: 10.1186/s12937-019-0515-6 (PMC6953463; doi:10.1186/s12937-019-0515-6)
Supplement: Supplementary file 2 — Additional file 2: Table S1. Baseline characteristics of participants and those dropping out of the prospective study (n = 6697). [file 12937_2019_515_MOESM2_ESM.docx]

Supplementary Table 1. Baseline characteristics of participants and those dropping out of the prospective study (n = 6,697)

|  | Participants | | Dropouts | | *P* ^a^ |
| --- | --- | --- | --- | --- | --- |
| Categorical variables | N | % | N | % |  |
| Number of subjects | 4,701 |  | 1,996 |  |  |
| Area (Shizuoka) | 2,606 | 55.4 | 972 | 48.7 | < 0.001 |
| Sex (women) | 2,237 | 47.6 | 990 | 49.6 | 0.131 |
| Current worker | 3,527 | 75.0 | 1,460 | 73.1 | 0.107 |
| Smoking |  |  |  |  | < 0.001 |
| never | 2,781 | 59.2 | 1,108 | 55.5 |  |
| former | 1,309 | 27.8 | 503 | 25.2 |  |
| current < 20 cigarettes/day | 313 | 6.7 | 183 | 9.2 |  |
| current 20-39 cigarettes/day | 279 | 5.9 | 183 | 9.2 |  |
| current ≥ 40 cigarettes/day | 19 | 0.4 | 19 | 1.0 |  |
| Drinking |  |  |  |  | 0.037 |
| never | 1,924 | 40.9 | 796 | 39.9 |  |
| former | 51 | 1.1 | 31 | 1.6 |  |
| current < 150 g/week | 1,781 | 37.9 | 719 | 36.0 |  |
| current 150-300 g/week | 562 | 12.0 | 250 | 12.5 |  |
| current ≥ 300 g/week | 383 | 8.1 | 200 | 10.0 |  |
| GHQ score at baseline |  |  |  |  | < 0.001 |
| 0 | 1,710 | 36.4 | 672 | 33.7 |  |
| 1 | 1,409 | 30.0 | 586 | 29.4 |  |
| 2 | 957 | 20.4 | 389 | 19.5 |  |
| 3 | 625 | 13.3 | 349 | 17.5 |  |
| Continuous variables | Mean | SD | Mean | SD |  |
| Age (years) | 53.3 | 9.2 | 51.8 | 10.1 | < 0.001 |
| Sleeping time (h/day) | 6.6 | 0.9 | 6.6 | 1.0 | 0.241 |
| Leisure-time exercise (METs･h/day) | 2.1 | 2.9 | 1.9 | 2.7 | < 0.001 |
| Eating breakfast (times/week) | 6.6 | 1.3 | 6.4 | 1.6 | < 0.001 |

GHQ: General Health Questionnaire; METs: metabolic equivalents; SD: standard deviation.

^a^ Categorical variables: χ^2^ test; continuous variables: *t*-test.
