# Supplementary material for: Associations between diet and mental health using the 12-item General Health Questionnaire: cross-sectional and prospective analyses from the Japan Multi-Institutional Collaborative Cohort Study
Source: Nutr J. 2020 Jan 9;19:2. doi: 10.1186/s12937-019-0515-6 (PMC6953463; doi:10.1186/s12937-019-0515-6)
Supplement: Supplementary file 3 — Additional file 3: Table S2. Cutoff values for quartiles of daily food and nutrient intakes at baseline. [file 12937_2019_515_MOESM3_ESM.docx]

Supplementary Table 2. Cutoff values for quartiles of daily food and nutrient intakes at baseline

|  | Cross-sectional study participants n = 9298 | | | | |  | Prospective study participants n = 4701 | | | | |
| --- | --- | --- | --- | --- | --- | --- | --- | --- | --- | --- | --- |
|  | Mean | SD | Q1/Q2 | Q2/Q3 | Q3/Q4 |  | Mean | SD | Q1/Q2 | Q2/Q3 | Q3/Q4 |
| Food groups (g/1000 kcal) |  |  |  |  |  |  |  |  |  |  |  |
| Fish | 29.1 | 14.8 | 18.4 | 26.7 | 36.1 |  | 29.7 | 14.8 | 18.8 | 27.6 | 36.6 |
| Meat and chicken | 22.4 | 12.6 | 14.1 | 19.5 | 28.9 |  | 21.9 | 12.7 | 13.7 | 18.9 | 28.2 |
| Dairy products | 75.3 | 62.5 | 22.4 | 65.6 | 108.7 |  | 77.7 | 62.9 | 25.0 | 68.3 | 112.7 |
| Vegetables | 82.3 | 50.2 | 47.8 | 71.4 | 103.8 |  | 83.1 | 49.7 | 48.5 | 71.9 | 104.9 |
| Nutrients |  |  |  |  |  |  |  |  |  |  |  |
| Protein (g/1000 kcal) | 31.3 | 4.5 | 28.2 | 31.0 | 33.9 |  | 31.3 | 4.5 | 28.3 | 31.0 | 33.9 |
| Fat (g/1000 kcal) | 26.4 | 6.8 | 21.7 | 26.0 | 30.7 |  | 26.1 | 6.7 | 21.4 | 25.7 | 30.3 |
| Carbohydrate (g/1000 kcal) | 140 | 15 | 131 | 141 | 150 |  | 140 | 15 | 132 | 142 | 150 |
| Calcium (mg/1000 kcal) | 314 | 96 | 244 | 302 | 369 |  | 316 | 96 | 245 | 304 | 371 |
| Vitamin B_1_ (mg/1000 kcal) | 0.396 | 0.083 | 0.337 | 0.392 | 0.446 |  | 0.390 | 0.082 | 0.331 | 0.385 | 0.440 |
| Vitamin B_2_ (mg/1000 kcal) | 0.661 | 0.172 | 0.541 | 0.647 | 0.767 |  | 0.665 | 0.170 | 0.548 | 0.650 | 0.770 |
| Vitamin D (μg/1000 kcal) | 4.26 | 1.81 | 2.91 | 3.97 | 5.13 |  | 4.35 | 1.84 | 2.95 | 4.08 | 5.22 |
| Carotene (μg/1000 kcal) | 1917 | 901 | 1300 | 1720 | 2287 |  | 1923 | 896 | 1310 | 1732 | 2297 |
| Saturated fatty acids (g/1000 kcal) | 6.86 | 1.79 | 5.56 | 6.69 | 7.92 |  | 6.80 | 1.79 | 5.50 | 6.63 | 7.85 |
| Monounsaturated fatty acids (g/1000 kcal) | 9.75 | 2.55 | 7.96 | 9.48 | 11.15 |  | 9.59 | 2.47 | 7.85 | 9.33 | 10.95 |
| n-6 polyunsaturated fatty acids (g/1000 kcal) | 6.58 | 1.82 | 5.32 | 6.37 | 7.60 |  | 6.50 | 1.77 | 5.27 | 6.30 | 7.47 |
| n-3 polyunsaturated fatty acids (g/1000 kcal) | 1.34 | 0.35 | 1.10 | 1.30 | 1.53 |  | 1.33 | 0.34 | 1.10 | 1.29 | 1.51 |
| n-3 highly-polyunsaturated fatty acids (g/1000 kcal) | 0.421 | 0.179 | 0.287 | 0.395 | 0.505 |  | 0.428 | 0.182 | 0.290 | 0.405 | 0.514 |

SD: standard deviation; Q1–Q4: quartiles 1–4.
