# Supplementary material for: Associations between diet and mental health using the 12-item General Health Questionnaire: cross-sectional and prospective analyses from the Japan Multi-Institutional Collaborative Cohort Study
Source: Nutr J. 2020 Jan 9;19:2. doi: 10.1186/s12937-019-0515-6 (PMC6953463; doi:10.1186/s12937-019-0515-6)
Supplement: Supplementary file 4 — Additional file 4: Table S3. Daily intakes of nutrients measured at follow-up (n = 4701). [file 12937_2019_515_MOESM4_ESM.docx]

Supplementary Table 3. Daily intakes of nutrients measured at follow-up (n = 4,701)

|  | Mean | SD | Cutoff values for quartiles (Q1–Q4) after energy adjustment (/1000 kcal) | | | Pearson’s correlation coefficient^a^ |
| --- | --- | --- | --- | --- | --- | --- |
|  |  |  | Q1/Q2 | Q2/Q3 | Q3/Q4 |  |
| Nutrients |  |  |  |  |  |  |
| Protein (g) | 52.6 | 10.7 | 28.5 | 31.3 | 34.5 | 0.538 |
| Fat (g) | 44.5 | 10.9 | 22.0 | 26.8 | 31.9 | 0.613 |
| Carbohydrate (g) | 234 | 63 | 130 | 140 | 149 | 0.643 |
| Calcium (mg) | 536 | 152 | 253 | 315 | 390 | 0.628 |
| Vitamin B_1_ (mg) | 0.657 | 0.087 | 0.344 | 0.398 | 0.457 | 0.617 |
| Vitamin B_2_ (mg) | 1.111 | 0.265 | 0.552 | 0.665 | 0.788 | 0.591 |
| Vitamin D (μg) | 7.15 | 3.26 | 2.94 | 4.04 | 5.25 | 0.542 |
| Carotene (μg) | 3244 | 1461 | 1332 | 1787 | 2411 | 0.598 |
| Saturated fatty acids (g) | 11.46 | 2.76 | 5.67 | 6.79 | 8.16 | 0.644 |
| Monounsaturated fatty acids (g) | 16.33 | 3.86 | 8.04 | 9.66 | 11.59 | 0.532 |
| n-6 polyunsaturated fatty acids (g) | 10.75 | 2.82 | 5.32 | 6.34 | 7.62 | 0.502 |
| n-3 polyunsaturated fatty acids (g) | 2.20 | 0.54 | 1.10 | 1.31 | 1.54 | 0.498 |
| n-3 highly-polyunsaturated fatty acids (g) | 0.701 | 0.320 | 0.288 | 0.400 | 0.514 | 0.519 |

SD: standard deviation; Q1–Q4: quartiles 1–4.

^a^ Correlation coefficients between energy-adjusted intake of each nutrient at baseline and that at follow-up.

All two-sided *P* values for the coefficients were less than 0.01.
