# Supplementary material for: Associations between diet and mental health using the 12-item General Health Questionnaire: cross-sectional and prospective analyses from the Japan Multi-Institutional Collaborative Cohort Study
Source: Nutr J. 2020 Jan 9;19:2. doi: 10.1186/s12937-019-0515-6 (PMC6953463; doi:10.1186/s12937-019-0515-6)
Supplement: Supplementary file 5 — Additional file 5: Table S4. Prospective logistic regression analyses of association between calcium/SFAs and GHQ-12 score with adjustment for dairy products. [file 12937_2019_515_MOESM5_ESM.docx]

Supplementary Table 4. Prospective logistic regression analyses of association between calcium/SFAs and GHQ-12 score*

|  |  | Participants with GHQ score ≥4 (n) | Model 3  (OR, 95% CI)a | | *P*-trend |
| --- | --- | --- | --- | --- | --- |
| Calcium | Q1 (low) | 211 | 1 | (Ref.) | 0.757 |
|  | Q2 | 203 | 0.85 | (0.65-1.11) |  |
|  | Q3 | 194 | 0.95 | (0.68-1.33) |  |
|  | Q4 (high) | 175 | 1.02 | (0.66-1.56) |  |
| Saturated fatty acids | Q1 (low) | 208 | 1 | (Ref.) | 0.267 |
|  | Q2 | 216 | 0.98 | (0.76-1.25) |  |
|  | Q3 | 176 | 0.87 | (0.65-1.17) |  |
|  | Q4 (high) | 183 | 0.84 | (0.59-1.19) |  |

* Adjusted for dairy products

GHQ: General Health Questionnaire; CI: confidence interval; OR: odds ratio; Q1–Q4: quartiles 1–4;

Odds ratios shown by quartile of intake (n = 4,701).

^a^ Model 3: adjusted for sex, age, area, employment, smoking, drinking, sleeping time, leisure time exercise, eating breakfast, total energy, and dairy products.

All variables (food and nutrient intakes and covariates) were measured at baseline.
