# Supplementary material for: Associations between diet and mental health using the 12-item General Health Questionnaire: cross-sectional and prospective analyses from the Japan Multi-Institutional Collaborative Cohort Study
Source: Nutr J. 2020 Jan 9;19:2. doi: 10.1186/s12937-019-0515-6 (PMC6953463; doi:10.1186/s12937-019-0515-6)
Supplement: Supplementary file 6 — Additional file 6: Table S5. Prospective logistic regression analyses for association between food/nutrient intakes and GHQ-12 score including those with GHQ-12 score ≥ 4 at baseline. [file 12937_2019_515_MOESM6_ESM.docx]

Supplementary Table 5. Prospective logistic regression analyses for association between food/nutrient intakes and GHQ-12 score*

|  |  | Participants with GHQ score ≥4 (n) | Model 2  (OR, 95% CI)a | | *P*-trend |
| --- | --- | --- | --- | --- | --- |
| Food groups |  |  |  |  |  |
| Fish | Q1 (low) | 450 | 1 | (Ref.) | 0.942 |
|  | Q2 | 419 | 0.88 | (0.75-1.04) |  |
|  | Q3 | 373 | 0.85 | (0.72-1.00) |  |
|  | Q4 (high) | 386 | 0.99 | (0.84-1.18) |  |
| Meat and chicken | Q1 (low) | 380 | 1 | (Ref.) | 0.712 |
|  | Q2 | 370 | 0.78 | (0.66-0.93) |  |
|  | Q3 | 399 | 0.80 | (0.67-0.95) |  |
|  | Q4 (high) | 479 | 0.89 | (0.75-1.06) |  |
| Dairy products | Q1 (low) | 436 | 1 | (Ref.) | < 0.001 |
|  | Q2 | 422 | 0.96 | (0.82-1.13) |  |
|  | Q3 | 386 | 0.78 | (0.66-0.92) |  |
|  | Q4 (high) | 384 | 0.75 | (0.63-0.89) |  |
| Vegetables | Q1 (low) | 396 |  | (Ref.) | 0.445 |
|  | Q2 | 427 | 1.04 | (0.88-1.22) |  |
|  | Q3 | 382 | 0.85 | (0.71-1.01) |  |
|  | Q4 (high) | 423 | 0.96 | (0.80-1.15) |  |
| Nutrients |  |  |  |  |  |
| Protein | Q1 (low) | 410 | 1 | (Ref.) | 0.099 |
|  | Q2 | 397 | 0.85 | (0.72-1.01) |  |
|  | Q3 | 386 | 0.74 | (0.62-0.88) |  |
|  | Q4 (high) | 435 | 0.86 | (0.72-1.03) |  |
| Carbohydrate | Q1 (low) | 450 | 1 | (Ref.) | 0.492 |
|  | Q2 | 422 | 1.02 | (0.86-1.20) |  |
|  | Q3 | 387 | 1.02 | (0.85-1.21) |  |
|  | Q4 (high) | 369 | 1.08 | (0.89-1.33) |  |
| Fat | Q1 (low) | 355 | 1 | (Ref.) | 0.723 |
|  | Q2 | 378 | 0.90 | (0.75-1.07) |  |
|  | Q3 | 419 | 0.88 | (0.73-1.06) |  |
|  | Q4 (high) | 476 | 0.95 | (0.78-1.15) |  |
| Calcium | Q1 (low) | 440 | 1 | (Ref.) | < 0.001 |
|  | Q2 | 392 | 0.75 | (0.63-0.89) |  |
|  | Q3 | 402 | 0.73 | (0.61-0.87) |  |
|  | Q4 (high) | 394 | 0.67 | (0.55-0.81) |  |
| Vitamin B_1_ | Q1 (low) | 346 | 1 | (Ref.) | 0.363 |
|  | Q2 | 385 | 0.97 | (0.80-1.18) |  |
|  | Q3 | 430 | 0.99 | (0.80-1.23) |  |
|  | Q4 (high) | 467 | 1.09 | (0.85-1.40) |  |
| Vitamin B_2_ | Q1 (low) | 438 | 1 | (Ref.) | 0.001 |
|  | Q2 | 381 | 0.75 | (0.63-0.89) |  |
|  | Q3 | 415 | 0.77 | (0.65-0.93) |  |
|  | Q4 (high) | 394 | 0.70 | (0.58-0.85) |  |
| Vitamin D | Q1 (low) | 429 | 1 | (Ref.) | 0.845 |
|  | Q2 | 446 | 0.98 | (0.83-1.15) |  |
|  | Q3 | 375 | 0.87 | (0.74-1.03) |  |
|  | Q4 (high) | 378 | 1.00 | (0.84-1.19) |  |
| Carotene | Q1 (low) | 389 | 1 | (Ref.) | 0.340 |
|  | Q2 | 440 | 1.05 | (0.89-1.25) |  |
|  | Q3 | 375 | 0.81 | (0.67-0.97) |  |
|  | Q4 (high) | 424 | 0.95 | (0.79-1.15) |  |
| SFA | Q1 (low) | 361 | 1 | (Ref.) | 0.020 |
|  | Q2 | 421 | 1.01 | (0.84-1.20) |  |
|  | Q3 | 398 | 0.79 | (0.65-0.96) |  |
|  | Q4 (high) | 448 | 0.82 | (0.67-1.01) |  |
| MUFA | Q1 (low) | 342 | 1 | (Ref.) | 0.116 |
|  | Q2 | 375 | 0.94 | (0.79-1.13) |  |
|  | Q3 | 427 | 1.03 | (0.85-1.23) |  |
|  | Q4 (high) | 484 | 1.12 | (0.92-1.36) |  |
| n-6 PUFA | Q1 (low) | 349 | 1 | (Ref.) | 0.472 |
|  | Q2 | 390 | 0.97 | (0.81-1.15) |  |
|  | Q3 | 424 | 1.01 | (0.84-1.21) |  |
|  | Q4 (high) | 465 | 1.05 | (0.87-1.26) |  |
| n-3 PUFA | Q1 (low) | 364 | 1 | (Ref.) | 0.010 |
|  | Q2 | 385 | 0.99 | (0.83-1.18) |  |
|  | Q3 | 412 | 1.04 | (0.87-1.25) |  |
|  | Q4 (high) | 467 | 1.24 | (1.03-1.49) |  |
| n-3 HUFA | Q1 (low) | 429 | 1 | (Ref.) | 0.896 |
|  | Q2 | 442 | 0.96 | (0.82-1.14) |  |
|  | Q3 | 370 | 0.87 | (0.73-1.03) |  |
|  | Q4 (high) | 387 | 1.03 | (0.86-1.23) |  |

* Includes those with GHQ-12 score ≥ 4 at baseline

GHQ: General Health Questionnaire; CI: confidence interval; OR: odds ratio; Q1–Q4: quartiles 1–4; SFA: saturated fatty acids; MUFA: monounsaturated fatty acids; PUFA: polyunsaturated fatty acids; HUFA: highly-polyunsaturated fatty acids.

Odds ratios shown by quartile of intake (n = 6,398).

^a^ Model 2: adjusted for sex, age, area, employment, smoking, drinking, sleeping time, leisure time exercise, eating breakfast, and total energy.

All variables (food and nutrient intakes and covariates) were measured at baseline.
